# Management of acute diverticulitis in Stage 0-IIb: indications and risk factors for failure of conservative treatment in a series of 187 patients

**DOI:** 10.1038/s41598-024-51526-5

**Published:** 2024-01-17

**Authors:** Amedea L. Agnes, Annamaria Agnes, Marta Di Grezia, Mauro Giambusso, Eleonora Savia, Michele Grieco, Valerio Cozza, Sabina Magalini, Gabriele Sganga

**Affiliations:** 1 Catholic University of the Sacred Heart, Largo F. Vito, 1, 00168 Rome, Italy; 2 Gemelli University Hospital Foundation and IRCCS, Largo A. Gemelli, 8, 00168 Rome, Italy; 3 S. Eugenio Hospital, Piazzale dell′Umanesimo, 10, 00144 Rome, Italy

**Keywords:** Intestinal diseases, Colonic diseases, Colitis

## Abstract

Left-sided acute diverticulitis in WSES Stage 0-IIb preferentially undergoes conservative management. However, there is limited understanding of the risk factors for failure of this approach. The aim of this study was to investigate the factors associated with the decision to perform conservative treatment as well as the predictors of its failure. We included patients with a diagnosis of WSES diverticulitis CT-driven classification Stage 0-IIb treated in the Emergency Surgery Unit of the Agostino Gemelli University Hospital Foundation between 2014 and 2020. The endpoints were the comparison between the characteristics and clinical outcomes of acute diverticulitis patients undergoing conservative versus operative treatment. We also identified predictors of conservative treatment failure. A set of multivariable backward logistic analyses were conducted for this purpose. The study included 187 patients. The choice for operative versus conservative treatment was associated with clinical presentation, older age, higher WSES grade, and previous conservative treatment. There were 21% who failed conservative treatment. Of those, major morbidity and mortality rates were 17.9% and 7.1%, respectively. A previously failed conservative treatment as well as a greater WSES grade and a lower hemoglobin value were significantly associated with failure of conservative treatment. WSES classification and hemoglobin value at admission were the best predictors of failure of conservative treatment. Patients failing conservative treatment had non-negligible morbidity and mortality. These results promote the consideration of a combined approach including baseline patients’ characteristics, radiologic features, and laboratory biomarkers to predict conservative treatment failure and therefore optimize treatment of acute diverticulitis.

## Introduction

Left-sided colonic complicated acute diverticulitis (AD) is a common clinical entity with rising prevalence, healthcare costs, and societal impact in industrialized countries^1–3^. The prevalence of diverticulosis in the western world is currently estimated at around 10% in 40-year-old patients and is increasing at more than 50% in > 60-year-old ones^4^. Among patients with diverticulosis, up to 7% will develop AD. Of these, about one quarter will be complicated^5–7^. Uncomplicated diverticulitis is usually treated conservatively whereas complicated diverticulitis is treated with percutaneous abscess drainage or operative intervention consisting of emergency resection with or without anastomosis^8^. Recently, there has been a strong tendency towards the conservative treatment of diverticulitis resulting in an expansion of knowledge about conservative treatment options for patients with WSES Stage 0-IIb. There have also been important developments of new treatment strategies for patients with Stage III and IV disease^9–12^. The conservative management of acute diverticulitis currently includes a wide range of treatment options: observation, administration of antibiotics covering gram-negative and anaerobic bacteria for complicated AD, and source control via percutaneous drain. Surgery is indicated in patients with Ib-II diverticulitis who have exhausted conservative management options without improvements in symptoms or remain systemically unwell^13^.

It is well-understood that disease severity might considerably vary in Stage 0-IIb diverticulitis. However, there is still limited understanding of those factors that can stratify the prognosis of patients with these stages of AD. There is a need for criteria that identify patients more likely to succeed with conservative treatment as well as guidelines for best management practices^14^.

Therefore, the aim of this study was to present our experience in the treatment of acute diverticulitis in WSES Stage 0 to IIb. Outcomes were factors associated with the decision to pursue upfront surgical treatment versus conservative treatment while comparing the outcomes of operative and conservative treatment for AD and identifying failure predictors of conservative therapy.

## Methods

### Patient selection and data collection

We included all patients with a diagnosis of acute diverticulitis treated in the Emergency Surgery Unit of Agostino Gemelli University Hospital Foundation between January 2014 and April 2020. We selected only stage 0-IIb patients classified according to the revised CT driven classification of left colon acute diverticulitis proposed by the WSES in 2015^15^ (Table 1s). We then introduced and confirmed the 2016 and 2020 WSES guidelines for the management of acute colonic diverticulitis in emergency settings^9,10^. Patients with a missing criterion of incomplete medical records and Stage III-IV diverticulitis were excluded.

**Table 1:** Clinicopathologic characteristics of patients in the operative and conservative treatment group.

Variable	Operative treatment n = 44 (%)	Conservative treatment n = 143 (%)	*p*-value
Age (years) mean ± SD	66 ± 14	58 ± 14	< 0.001
Gender n (%)			
Male	27 (61.4)	80 (55.9)	0.525
Female	17 (38.6)	63 (44.1)	
Charlson Index mean ± SD	4.12 ± 3.89	2.11 ± 2.23	< 0.001
Shock n (%)			
No	35 (79.5)	121 (98.6)	< 0.001
Yes	9 (20.5)	2 (1.4)	
Positive Blumberg sign n (%)**			
No	22 (61.4)	114 (79.7)	0.013
Yes	11 (38.6)	29 (20.3)	
Glycemia mean ± SD	125 ± 35	111 ± 33	0.017
Creatinine mean ± SD	1.14 ± 1.57	0.98 ± 0.73	0.349
Hemoglobin mean ± SD	12.99 ± 1,99	15,04 ± 18,36	0.467
WBC mean ± SD	13,50 ± 5,53	13,25 ± 4,13	0.785
PLT mean ± SD	307 ± 289	267 ± 104	0.375
Abs neutrophil count	11.36 ± 5.12	10.52 ± 4.01	0.325
Abs lymph count	1.43 ± 0.84	1.80 ± 0.90	0.017
N/L ratio	10.40 ± 8.22	7.49 ± 6.57	0.038
P/L ratio	327.78 ± 574.40	267.21 ± 104.87	0.099
Previous conservative treatment n (%)			
No	33 (75)	127 (88.8)	0.023
Yes	11 (25)	16 (11.2)	
WSES Stage n (%)			
Stage 0	0 (0)	12 (8.4)	< 0.001
Stage Ia	7 (15.9)	55 (38.5)	
Stage Ib	9 (20.5)	39 (27.3)	
Stage IIa	12 (27.3)	22 (15.4)	
Stage IIb	16 (36.4)	15 (10.5)	
Sigmoid thickening n (%)			
No	5 (11.4)	3 (2.1)	0.019
Yes	39 (88.6)	140 (97.9)	
Pericolic collection n (%)			
No	21 (47.7)	79 (52.2)	0.382
Yes	23 (52.3)	74 (44.8)	
Pelvic fluid n (%)^a^			
No	23 (53.5)	91 (64.5)	0.191
Yes	20 (46.5)	50 (35.5)	
Extrapelvic fluid n (%)^b^			
No	35 (85.4)	128 (90.1)	0.399
Yes	6 (14.6)	14 (9.9)	
Pericolic air n (%)^c^			
No	19 (45.2)	68 (47.9)	0.763
Yes	23 (54.8)	74 (52.1)	
Distant air n (%)^d^			
No	27 (62.8)	129 (90.8)	< 0.001
Yes	16 (37.2)	13 (9.2)	

^a^3 missing cases, ^b^4 missing cases, ^c^3 missing cases, ^d^2 missing cases.

Data were retrospectively extracted from the medical records and included date of treatment, age, gender, Charlson Comorbidity Index, laboratory values at admission (glycemia, creatinine, BUN, Ca, Na, K, amylase, lactate-dehydrogenase, GPT, bilirubinemia, INR, Hb, WBC, platelet count), history of previous conservative treatment for diverticulitis, radiologic features at the admission CT (sigmoid thickening, pericolic collection, dimension of the collection, pelvic fluid, extra-pelvic fluid, pericolic free air, diffuse free air), WSES grade, choice of conservative or operative treatment, administration of antibiotics, percutaneous drain, type of surgery, complications associated to surgery or conservative treatment, length of stay, readmission, or recurrent diverticulitis within 30 days. Surgical complications were graded according to the Clavien-Dindo classification^16^. Major complications were defined as failure of the conservative treatment or mortality for patients treated conservatively or with Clavien-Dindo grade III-V for patients undergoing operative treatment.

The research was performed in accordance with the Declaration of Helsinki.

### Treatment of acute diverticulitis

Patients came to our attention through the Emergency Department of our Institution or during hospital stays for other reasons. Patients with suspect AD underwent an abdominopelvic CT-scan and were classified according to the WSES classification. Patients with AD were treated with bowel rest and antibiotic therapy consisting of a piperacillin/tazobactam regimen or an aminoglycoside plus metronidazole regimen. In cases of presentation with fever > 38° or sepsis, patients were treated according to internal critical care guidelines with targeted antibiotics or empiric therapy with meropenem. Patients underwent a percutaneous drainage of localized collections greater than 5 cm. Poor or no response to medical treatment was established after 3–4 days of care without clinical improvement or in the case of worsening laboratory values or clinical conditions at any timepoint.

### Study endpoints and outcome measures

The primary endpoint of this study is a comparison between the characteristics and clinical outcomes of patients with AD undergoing conservative or operative treatment. The secondary endpoint is the identification of predictors of failure of conservative treatment across the entire population and in the subgroup of patients with Ib-IIb diverticulitis. Failure of conservative treatment was considered as the need to perform any surgical intervention during the same admission or as a readmission to ours or another hospital with recurrent diverticulitis within 30 days.

### Statistics

Clinicopathological characteristics were summarized using frequencies and percentages for categorical variables; means and standard deviations or medians and ranges were used for continuous variables. Patients’ characteristics were compared using the Pearson’s Chi-squared test or the Fisher’s exact test for categorical variables and the Student’s t-tests for continuous variables. Multivariable analyses for variables associated with the primary and secondary outcomes were conducted with a backward logistic regression method using *p* < 0.1 as the limit for inclusion in the progressive steps of the regression model. Statistical analysis was conducted using SPSS Statistics for Macintosh, Version 25.0. (IBM Corp, Armonk, NY). All statistical tests were 2-sided at a significance level of *p* < 0.05.

### Ethics approval and consent to participate

All the study was based on hospital data obtained consulting clinical records; therefore, ethical disclosure was not necessary.

## Results

Between January 2014 and July 2020, 220 patients with AD were treated in our Institution. Of those, 10 and 18 presented with Stage III and IV diverticulitis, respectively; five patients had incomplete medical records. After the application of the inclusion and exclusion criteria, 187 patients remained eligible for the study. Of these, the mean age was 60 ± 15, and the male/female ratio was 1.34:1. Patients who were candidates to operative or conservative treatment were 44 (23.2%) and 143 (76.8%), respectively (Table 1). Two patients were already hospitalized for another reason at the time of evaluation. Of these two, one underwent operative treatment and the other was a candidate for conservative treatment.

### Indications and outcomes associated with operative versus conservative treatment

The multivariable logistic regression identified increased age (*p* = 0.017), the presence of hemodynamic instability (*p* = 0.033), a higher WSES diverticulitis grade (>/= IIa) (*p* = 0.003 and *p* < 0.001, respectively), and a previous episode of AD (*p* = 0.020) as variables associated with operative treatment. Patients undergoing conservative treatment, patients undergoing operative treatment had a longer length of stay (mean 15 vs 7 days, *p* = 0.001), a higher rate of mortality (9.1% vs 1.4%, *p* = 0.028), and a higher but non-significant rate of major complications (29.5% vs 20.4%, *p* = 0.206) and readmission (12.5% vs 7.7%, *p* = 0.351) (Tables 2 and 3).

**Table 2:** Multivariable analysis of factors associated with decision on the conservative treatment in patients with WSES Stage 0-IIb.

Variable	Beta coefficient	*p*-value	OR	CI 95% low	CI 95% high
Age	− 0.036	0.017	0.965	0.936	0.994
Shock	− 1.870	0.033	0.154	0.028	0.863
Stage IIa	− 1.487	0.003	0.226	0.085	0.603
Stage IIb	− 1.782	0.000	0.168	0.062	0.456
Previous conservative treatment	− 1.194	0.020	0.303	0.111	0.829
Constant	4.551	0.000	94.683		

Variable(s) entered on step 1: age, gender, Charlson Comorbility Index, shock, Blumberg sign, WBC, thickening of the sigmoid colon, pericolic collections, pelvic ascites, extra-pelvic ascites, pericolic free air, pneumoperitoneum, WSES Stage (0, Ia, Ib, IIa, IIb), previous conservative treatment.

**Table 3:** Clinical outcomes associated with operative versus conservative treatment in 187 patients.

Variable	n = 44 (%)	n = 143 (%)	*p*-value
Length of stay (mean ± SD)	14.93 ± 16.15	7.41 ± 11.21	0.001
Major complications n (%)*			
No	31 (70.5)	113 (79.6)	0.206
Yes	13 (29.5)	29 (20.4)	
Readmission n (%)			
No	35 (87.5)	131 (93.43)	0.351
Yes	5 (12.5)	11 (7.7)	
Mortality**			
No	40 (90.9)	141 (98.6)	0.028
Yes	4 (9.1)	2 (1.4)	

*missing: 1 patient, **one patient in the conservative group died after readmission and emergency reoperation.

### Outcomes of primary versus secondary operative treatment

Of the 143 patients who were candidates for conservative treatment, 12 (8.4%) underwent the placement of a percutaneous drain. Overall, 30 patients failed conservative treatment (21%). The hospitalized patient was among those failing conservative treatment. Specifically, one patient had recurrent diverticulitis (within 30 days) treated conservatively, another had recurrent diverticulitis and was treated elsewhere, and 28 patients underwent emergent surgical treatment at our institution (Tables 2s and 3s).

### Predictors of failure of conservative treatment in the whole population and in patients with Stage Ib-IIb diverticulitis

Multivariable logistic regression on all 143 patients who were candidates for conservative treatment identified variables associated with the failure of operative treatment (Table 4): a lower hemoglobin at the first evaluation (*p* = 0.001), a WSES Stage greater than Ia (*p* = 0.007 for Stage Ib, *p* = 0.004 for Stage IIa and *p* = 0.001 for Stage Iib, respectively), a positive Blumberg sign at the first evaluation (*p* = 0.044), and a previous episode of AD treated conservatively (*p* = 0.033).

**Table 4:** Multivariable analysis of factors associated with failure of the conservative treatment in all patients.

Variable	Beta coefficient	*p*-value	OR	CI 95% low	CI 95% high
Glycemia	− 0.019	0.096	0.981	0.959	1.003
Hemoglobin	− 0.588	0.001	0.555	0.396	0.779
WSES Stage Ib	1.978	0.007	7.231	1.705	30.666
WSES Stage IIa	2.404	0.004	11.068	2.153	56.888
WSES Stage IIb	3.206	0.001	24.671	3.772	161.381
Positive Blumberg sign	− 1.908	0.044	0.148	0.023	0.947
Previous conservative treatment	1.615	0.033	5.028	1.135	22.271
Constant	6.965	0.008	1059.329		

Variable(s) entered on step 1: age, gender, Charlson Comorbidity Index, previous conservative treatment, Blumberg sign, glycemia, creatinine, hemoglobin, WBC, ANC, ALC, N/L ratio, PLT, P/L ratio, thickening of the sigmoid colon, pericolic collections, pelvic ascites, extrapelvic ascites, pericolic free air, pneumoperitoneum, WSES Stage (0-Ia, Ib, IIa, IIb).

Among 76 Ib-IIb stage patients undergoing conservative treatment, the only predictor of failure of conservative treatment was a lower hemoglobin at the first evaluation (*p* = 0.007) (Table 5).

**Table 5:** Multivariable analysis of factors associated with failure of the conservative treatment in patients with WSES Stage Ib-IIb.

Variable	Beta coefficient	*p*-value	OR	CI 95% low	CI 95% high
Glycemia	− 0.028	0.069	0.973	0.944	1.002
Hemoglobin	− 0.462	0.007	0.630	0.450	0.882
N/L ratio	0.071	0.115	1.074	0.983	1.173
Positive Blumberg sign	− 1.884	0.093	0.152	0.017	1.368
Constant	8.092	0.004	3269.619		

Variable(s) entered on step 1: age, gender, Charlson Comorbidity Index, previous conservative treatment, Blumberg sign, glycemia, creatinine, hemoglobin, WBC, ANC, ALC, N/L ratio, PLT, P/L ratio, thickening of the sigmoid colon, pericolic collections, pelvic ascites, extrapelvic ascites, pericolic free air, pneumoperitoneum, WSES Stage (Ib, IIa, IIb).

## Discussion

This study evaluated a population of patients presenting with AD in WSES Stage 0-IIb. The choice for operative versus conservative treatment was associated with the clinical presentation, an older age, a higher diverticulitis grade, and previous conservative treatment; 21% of patients failed conservative treatment. While patients failing conservative treatment did not have greater morbidity or mortality than patients undergoing upfront operative treatment, the major morbidity of 17.9% and mortality of 7.1% in this category were not negligible. A previously failed conservative treatment as well as a greater diverticulitis stage and a lower hemoglobin value were significantly associated with failure of conservative treatment. In the subgroup of patients with WSES Ib-IIb disease, a lower hemoglobin value was the only independent factor associated with failure of the conservative treatment.

The treatment of acute diverticulitis in the emergency setting should strictly be dependent on the patient's abdominal exam and should include a comprehensive clinical picture. However, several surgeon-related factors are known to influence clinical decisions in this setting. The surgeon’s experience and intuition usually have a role, but this factor has been frequently associated with an overestimation of patient risk^17^. The IPOD study previously demonstrated how the management of AD is often guided by the personal preferences of the surgeon in an Italian surgical unit^18^. Moreover, the results of another Italian survey showed the low implementation of international guidelines in surgical practice in the management of acute left diverticulitis^19^. This study identified factors associated with a decision on upfront surgery instead of conservative treatment, thus highlighting how a critically ill condition, a higher age, a higher grade, as well as a history of previous conservative treatment were associated with the indications for upfront surgery. Almost all factors that seem to influence this decision were covered by WSES guidelines on AD management except for the presence of a previous episode of AD. Our results highlight the importance of applying standardized and updated institutional protocols for treating AD based on international recommendations^20^ to minimize the risk of selection bias and increase the reliability of future studies on outcome prediction.

The approach to conservative treatment for AD is evolving from the recommendation of not administering antibiotic therapy in lower grade patients^21,22^. There is also a consideration for an outpatient approach in low-risk cases^23^. The availability of several strategies to minimize the risk of surgical intervention or even resection when surgery for source control is required should also be considered (i.e., percutaneous drain, toilette + lavage). There is a well-known difficulty in identifying predictors of failure of conservative therapy in acute diverticulitis and hence stratifying patients for conservative treatment, but this topic is not broadly covered even in the most recent WSES guidelines.

Only few studies have investigated this topic in recent years and have reported a substantial rate of failure of conservative treatment depending on AD grade. Several studies have found radiologic features to be better predictors of treatment outcome even though most have been limited to Stage I patients. Colas et al. studied 91 patients affected by AD with extraluminal air at the admission CT-scan and described a failure rate of conservative treatment of 31.9%. They found that independent risk factors for failure were a greater diameter of the pneumoperitoneum (> 5 mm) (OR = 5.193; *p* = 0.015) and the presence of peritoneal fluid in the pouch of Douglas (OR = 4.103; *p* = 0.036)^24^. Another study by Titos-Garcia et al. conducted on 77 patients suffering from uncomplicated AD with extraluminal air at the admission CT-scan found that the failure rate of conservative treatment was 15.6%. An ASA grade III-IV (OR = 5.49; CI 95%, 1.04–29.07) and a distant free air location (OR = 4.81; CI 95%, 1.03–22.38) were the only two independent factors associated with treatment failure^25^. In a systematic review of the literature on the same topic, failure was associated with a higher volume of free air, distant free air, and the presence of an abscess among 407 patients with AD undergoing conservative treatment^26^. Our research confirmed the validity of the WSES CT driven classification in terms of prediction of treatment failure in agreement with previous studies highlighting radiologic features as independent predictors of failure^27^. In our study however, radiologic characteristics outside the WSES classification were not significantly associated with failure. Future work should use more advanced imaging-based approaches. The use of radiomics^28^ could improve the predictive value of the initial CT-scan based on WSES classification and quantitative feature selection analyzed via machine-learning algorithms.

In other studies, biomarkers were identified as effective predictors of treatment outcome. A 2019 study by Bolkenstein et al. focused on a population of 109 Stage Ia diverticulitis patients and investigated predictors of failure of non-operative management that was defined as the need for percutaneous abscess drainage or non-elective surgery within 30 days after the first presentation. These cases had a failure rate of 8%. An increased CRP level (> 170 mg/dl) at presentation was the only independent predictor for treatment failure^29^. CRP was not available in our study. However, the use of sole CRP at admission has some theoretical limitations because CRP values might not yet be elevated because there is a delay of 6–8 h from the onset of the disease and CRP peaks at 48 h^30^. Moreover, a single CRP value instead of its variation might be less reproducible because there are known high- and low- CRP expressors^31^. Ahmadi et al. found that the trend of CRP is effective in predicting the failure of conservative treatment because patients with low CRP but a rapid rise at 48 h (from < 80 mg/dl to > 200 mg/dl) or with a high CRP at admission (> 80 mg/dl) and after 48 h (> 200 mg/dl) have a higher risk of treatment failure^32^. Rather, our study found that a lower hemoglobin at admission was a predictor of failure of conservative treatment. Notably, the hemoglobin value outperformed each of the available inflammatory biomarkers (WBC, N/L ratio, P/L ratio) as a predictor of failure. Accordingly, anemia is known to be strictly associated with inflammatory states^33^, to be a negative prognostic factor for infective events associated with the treatment of various conditions, and to be itself indirectly associated with chronic diseases as well as to worse nutritional status^34–36^. A recent study by Muse et al.^37^ found that the preoperative anemia of patients undergoing colectomy for diverticulitis in both elective and emergency setting increased the risk of complications, readmissions, and intra/postoperative blood transfusions. Hence, this factor appears to be associated with a higher risk of conservative treatment failure than surgical treatment; thus, further studies are needed to clarify this important correlation.

In our experience, failure of conservative therapy was associated with relevant morbidity and rate of stoma creation. Within the literature focused on the outcomes of conservative treatment, there are few reports on the surgical outcomes associated with failure of conservative treatment. Based on our results, we identified a subset of patients treated conservatively that have a risk of failure, and consequently, of poor surgical outcomes. For these patients, a more aggressive conservative treatment strategy (i.e., a lower dimensional threshold for percutaneous drainage or a more effective antibiotic therapy) might improve outcomes.

The main advantages of this study are the number of cases analyzed—this is one of the largest series in the literature analyzed to identify predictors of treatment failure in AD. The main limitations of this study are its retrospective and single center nature, which may have resulted in confounding bias when directing patients to upfront conservative versus operative treatment. Thus, one of our analyses was specifically directed at investigating possible confounding to contextualize the results. The analysis showed that almost all factors that were associated with the decision on conservative versus operative treatment were coherent with the WSES recommendations. Another possible bias of this study is the presence in the cohort of patients that were already hospitalized because those patients might have been frailer than the others^38^. However, patients who were already hospitalized represented a minority of the patients analyzed (2) and in this case their clinicopathological characteristics did not differ from the baseline of most patients. Moreover, to control confounding, we addressed for the Charlson Comorbidity Index in the multivariable analysis. Another limitation of the study was the incomplete evaluation of the CRP values at admission for all patients and incomplete data on the variation of the laboratory analysis once conservative therapy had been started. We did not have data on the specific costs associated with the different types of treatment and their possible failure, nor did we have details on the costs associated with the occurrence of complications or readmission.

Based on these results, several possible predictors of failure of the conservative treatment should be considered including patients’ conditions, laboratory biomarkers, and radiologic data. Moreover, the variations between laboratory biomarkers after the start of conservative treatment should also be followed to further investigate their significance. Composite scores accounting for all these predictors could be relevant for targeting treatment and should be the aim of future studies. Ideally, based on such parameters and within standardized protocols for treatment, one could generate a model predictive of failure of the conservative treatment for AD. This could help treat patients at risk of failure more aggressively to reduce the complications and costs associated with this event.

## Conclusions

We investigated the predictive factors for failure of conservative treatment, thus confirming radiologic findings (the WSES CT driven classification of left colon acute diverticulitis) as the best determinants for its failure. This data suggests that the use of biomarkers could be helpful in predicting the failure of conservative treatment for acute diverticulitis. A combined approach could have relevant advantages to direct personalized treatment, thus minimizing the risk of morbidity, mortality, readmission, and cost increase associated with treatment failure.

### Supplementary Information

Supplementary Tables.

## Data Availability

The datasets generated during and/or analyzed during the current study are not publicly available because they are based in consulting clinical records, but they are available from the corresponding author on reasonable request.

## Abbreviations

AD: Acute diverticulitis
CT: Computed tomography
WSES: World society of emergency surgery
