# Supplementary material for: Management of acute diverticulitis in Stage 0-IIb: indications and risk factors for failure of conservative treatment in a series of 187 patients
Source: Sci Rep. 2024 Jan 17;14:1501. doi: 10.1038/s41598-024-51526-5 (PMC10794215; doi:10.1038/s41598-024-51526-5)
Supplement: Supplementary file 1 — Supplementary Tables. [file 41598_2024_51526_MOESM1_ESM.docx]

**Supplementary tables**

| **Table 1s Revised CT scan classification of acute diverticultis [Sartelli M, Moore FA, Ansaloni L, Di Saverio S, Coccolini F, Griffiths EA, et al. A proposal for a CT driven classification of left colon acute diverticulitis. World J Emerg Surg. 2015;10:3.]** | |
| --- | --- |
| **Uncomplicated** | |
| Stage 0 | Diverticula, thickening of the colonic wall or increased density of the pericolic fat |
| **Complicated** | |
| Stage 1a | Pericolic air bubbles or little pericolic fluid without abscess (within 5 cm from inflamed bowel segment) |
| Stage 1b | Abscess ≤ 4 cm |
| Stage 2a | Abscess > 4 cm |
| Stage 2b | Distant air (>5 cm from inflamed bowel segment) |
| Stage 3 | Diffuse fluid without distant free air (no hole in colon) |
| Stage 4 | Diffuse fluid with distant free air (persistent hole in colon) |

| **Table 2s: Type of surgery associated with primary vs secondary treatment (failure) vs elective operative treatment** | | | | |
| --- | --- | --- | --- | --- |
| Variable | Primary operative treatment  n = 44 (%) | Secondary treatment/failure  n = 28 (%) | Elective surgery  n = 29 (%) | *p*-value |
| Sigmoidectomy without diverting stoma | 9 (20.5) | 8 (28.6) | 26 (89.7) | <0.001 |
| Sigmoidectomy with diverting stoma | 4 (9.1) | 4 (14.3) | 2 (6.9) | 0.628 |
| Primary anastomosis ± stoma | 13 (29.5) | 12 (42.9) | 28 (96.6) | <0.001 |
| Hartmann | 26 (59.1) | 11 (39.3) | 1 (3.4) | <0.001 |
| Toilette + drain | 2 (4.5) | 0 (0) | 0 (0) | 0.267 |
| Colostomy | 2 (4.5) | 5 (17.9) | 0 (0) | 0.021 |

| **Table 3s: Clinical outcomes associated with primary vs secondary (failure) operative treatment** | | | |
| --- | --- | --- | --- |
| Variable | Primary operative treatment  n = 44 (%) | Secondary treatment/failure  n = 28 (%) | *p*-value |
| Major surgical complications n (%)*  No  Yes | 31 (70.5)  13 (29.5) | 23 (82.1)  5 (17.9) | 0.264 |
| Readmission n (%)**  No  Yes | 35 (87.5)  5 (12.5) | 23 (89.3)  3 (10.7) | 0.907 |
| Mortality  No  Yes | 40 (90.9)  4 (9.1) | 26 (92.9)  2 (7.1) | 1 |

**excluding postoperative deaths during the same admission
